# Prognostic significance and therapeutic implications of redox metabolism-related genes in head and neck squamous cell carcinoma

**DOI:** 10.3389/ebm.2025.10623

**Published:** 2025-09-19

**Authors:** Lina Yang, Jingyu Hai, Jiayi Liu, Shaohua Shen, Lin Su, Juan Sun

**Affiliations:** ^1^ Department of Otolaryngology, Head and Neck Surgery, Inner Mongolia Medical University Affiliated Hospital, Hohhot, China; ^2^ Department of Chinese Traditional Medicine Department, Inner Mongolia Medical University Affiliated Hospital, Hohhot, China

**Keywords:** redox metabolism, immune cell infiltrations, head and neck squamous cell carcinomas, chemotherapy sensitivity, tumor microenvironment

## Abstract

Head and neck squamous cell carcinomas (HNSC) are associated with alterations in redox metabolism. This study aims to identify differentially expressed genes (DEGs) related to redox metabolism in HNSC and assess their prognostic values. We utilized the limma package for identifying redox metabolism-related DEGs and performed univariate and multivariate Cox regression analyses to evaluate their prognostic significance. Gene set variation analysis (GSVA), immune cell infiltration analysis, and single-cell RNA sequencing were utilized to explore the relationships between gene expression and tumor processes. Chemotherapy sensitivity was assessed based on *ERP44* expression levels. Additionally, pan-cancer analysis was conducted to evaluate *ERP44* expression and its prognostic value across different cancer types. The analysis identified several DEGs with significant prognostic value, including *ERP44*, which was significantly associated with poor prognosis in HNSC patients. High *ERP44* expression correlated with reduced overall survival, disease-specific survival, and progression-free interval. *ERP44* was notably overexpressed in tumor tissues and associated with key oncogenic pathways and immune cell infiltration patterns. Chemotherapeutic drug sensitivity analysis revealed that high *ERP44* expression increased sensitivity to Paclitaxel, Vinblastine, and Sorafenib but decreased sensitivity to Rapamycin. Pan-cancer analysis indicated that *ERP44* is differentially expressed and prognostic across multiple cancer types. Our findings highlight the crucial role of redox metabolism-related DEGs, particularly *ERP44*, in HNSC progression and prognosis. *ERP44* serves as a potential biomarker for prognosis and therapeutic response, warranting further research into its biological functions and potential as a therapeutic target.

## Impact statement

The primary objective of this study is to elucidate the role of redox metabolism-related DEGs in HNSCC, focusing on their prognostic value and clinical applicability. By correlating gene expression profiles with patient outcomes and treatment responses, this research aims to contribute to the field of precision oncology in HNSCC. Ultimately, the findings could pave the way for innovative therapeutic strategies targeting redox metabolism, enhancing the management of this challenging malignancy.

## Introduction

The majority of cancers located in the head and neck region originate from the mucosal epithelium found in the oral cavity, pharynx, and larynx, and are collectively referred to as head and neck squamous cell carcinoma (HNSC). HNSC is a prevalent malignancy with significant morbidity and mortality, accounting for a considerable portion of cancer-related deaths globally [1, 2]. It is characterized by its aggressive nature and the challenges associated with effective treatment options. Traditional therapeutic approaches, including surgery, radiation therapy, and chemotherapy, often face limitations due to tumor heterogeneity, resistance to treatment, and recurrence, necessitating a deeper understanding of the underlying molecular mechanisms driving HNSC progression and therapy response [3–5].

Recent advancements in the field of cancer research have significantly highlighted the critical and transformative role that molecular biomarkers play in not only improving prognosis but also in personalizing treatment strategies specifically for HNSC [6]. Among the various biomarkers under investigation, redox metabolism-related genes have garnered considerable attention due to their potential involvement in influencing tumor behavior and the overall response to therapeutic interventions [7, 8]. The dysregulation of redox homeostasis, which refers to the balance between oxidative and reductive processes within the cell, contributes to the accumulation of oxidative stress, a condition that is increasingly implicated in the progression of cancer and the development of resistance to various therapies [9–11]. Several comprehensive studies have identified specific redox-related genes that may serve as valuable prognostic biomarkers, thereby offering crucial insights into the complex interplay between oxidative stress, the tumor microenvironment, and the immune response, ultimately paving the way for more effective and tailored treatment approaches for patients suffering from this challenging disease [12, 13].

Despite extensive research efforts in the field, the specific mechanisms by which redox metabolism influences HNSC remain poorly understood, highlighting a significant gap in the current literature that urgently needs to be addressed. This lack of clarity underscores the critical need for the identification of novel biomarkers and therapeutic targets, which are essential for enhancing patient outcomes and improving treatment strategies. Furthermore, the intricate relationship between these genes and various clinical parameters, including immune cell infiltration and sensitivity to chemotherapy, has not been sufficiently explored, leaving a void in our understanding of their roles in disease progression and treatment response. To tackle these pressing gaps in knowledge, this study employs advanced bioinformatics approaches that utilize large-scale genomic datasets sourced from The Cancer Genome Atlas (TCGA) and the HNSC Clinical Proteomic Tumor Analysis Consortium (CPTAC). These methods facilitate the thorough analysis of extensive datasets, enabling researchers to identify differentially expressed genes (DEGs) that are intricately linked to redox metabolism in HNSC. The comprehensive nature of these analyses not only provides an opportunity to uncover novel insights into the prognostic significance of the identified genes but also sheds light on their potential associations with immune cell dynamics and responses to chemotherapy, ultimately paving the way for more effective therapeutic interventions.

**FIGURE 1:**
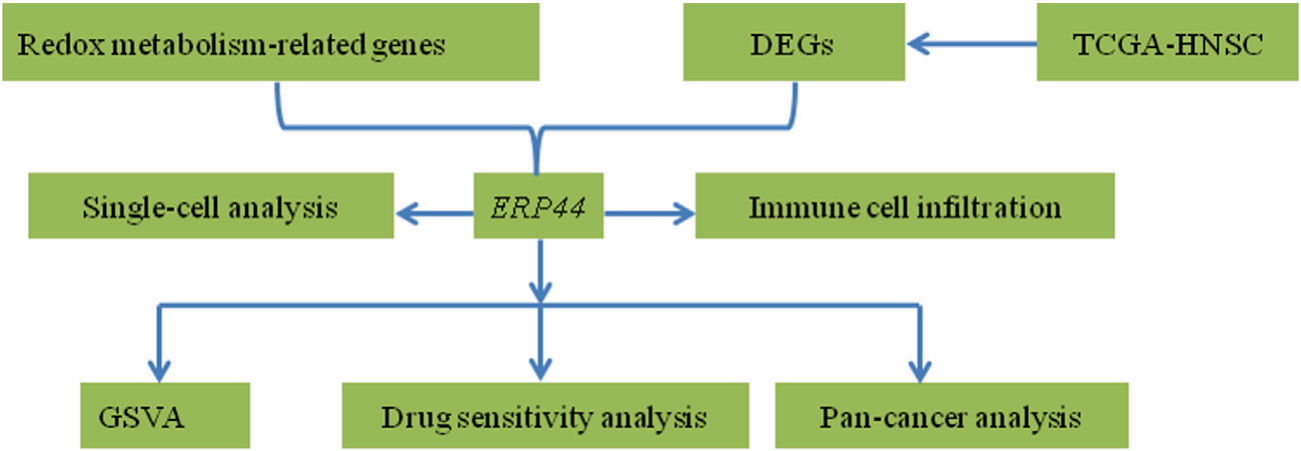
Abstract diagram of the study.

The primary objective of this study is to elucidate the role of redox metabolism-related DEGs in HNSC, focusing on their prognostic value and clinical applicability. By correlating gene expression profiles with patient outcomes and treatment responses, this research aims to contribute to the field of precision oncology in HNSC. Figure 1 presents the abstract diagram of this study. Ultimately, the findings could pave the way for innovative therapeutic strategies targeting redox metabolism, enhancing the management of this challenging malignancy.

## Materials and methods

### Data acquisition

The data used in this study were primarily sourced from the TCGA (The Cancer Genome Atlas)^1^ and HNSC-CPTAC databases.^2^ The TCGA-HNSC dataset comprises 548 tumor samples and 44 adjacent normal tissue samples. Detailed clinical baseline characteristics of the patient cohort are presented in Supplementary Table S1. RNA sequencing data, clinical information, and relevant survival data for HNSC patients were retrieved from TCGA using the TCGAbiolinks package in R. For the analysis of protein expression levels, data from the HNSC-CPTAC dataset were utilized.

### Identification of redox metabolism-related DEGs

Differential expression analysis of redox metabolism-related genes between normal and tumor samples in HNSC was performed using the limma package. A set of predefined redox metabolism-related genes was extracted from the GOBP_CELL_REDOX_HOMEOSTASIS gene set (obtained from the Molecular Signatures Database).^3^ From this gene set, differential expression was assessed with an adjusted p-value threshold of 0.05, yielding redox metabolism-related DEGs. The results were visualized using a heatmap generated with the pheatmap package. Genes exhibiting significant differential expression were further analyzed for their prognostic potential using univariate Cox regression analysis.

### Prognostic analysis

Univariate and multivariate Cox regression analyses were performed to evaluate the prognostic significance of the identified redox metabolism-related DEGs. Cox regression models were constructed using the survival package. The univariate analysis first identified individual prognostic markers with a significance threshold of p < 0.05, followed by multivariate Cox regression to assess the independent prognostic value of each gene. Forest plots were generated to illustrate hazard ratios (HRs) and their corresponding 95% confidence intervals (CIs). The prognostic significance of *ERP44* expression was analyzed using Kaplan-Meier survival analysis. The survminer package was used to assess the relationship between *ERP44* expression and various survival outcomes, including overall survival (OS), disease-specific survival (DSS), progression-free interval (PFI), and disease-free interval (DFI). Log-rank tests were applied to compare survival curves between high and low *ERP44* expression groups. Additionally, Wilcoxon rank-sum tests were conducted to compare *ERP44* expression levels between tumor and normal tissues in the TCGA-HNSC dataset.

### Gene set variation analysis (GSVA)

Gene Set Variation Analysis (GSVA) was performed to evaluate the association between *ERP44* expression and various oncogenic pathways in the TCGA-HNSC dataset. The GSVA package was used to calculate GSVA scores for 14 predefined hallmark pathways (MSigDB) related to angiogenesis, epithelial-mesenchymal transition (EMT), proliferation, hypoxia, and other tumor-related processes. Spearman’s correlation coefficients (|R| >0.10, FDR-adjusted p < 0.05) were calculated to assess the relationship between ERP44 expression and GSVA scores. Only pathways meeting both correlation strength (|R| >0.10) and statistical significance thresholds were reported in the main text.

### Immune cell infiltration analysis

The relative proportion of immune cell types in the TCGA-HNSC dataset was estimated using the ssGSEA algorithm. The correlation between *ERP44* expression and immune cell infiltration levels was assessed using the spearman’s correlation analysis (|R| > 0.10, FDR-adjusted p < 0.05). Differential immune cell infiltration between high and low *ERP44* expression groups (stratified by median expression) was evaluated, and statistical significance was determined using Wilcoxon rank-sum tests (p < 0.05).

### Single-cell RNA sequencing analysis

Single-cell RNA-sequencing data from the GSE103322 dataset (18 primary HNSC patients, 5,902 cells total) were analyzed using the Seurat package to investigate the expression of *ERP44* at the single-cell level. Uniform Manifold Approximation and Projection (UMAP) plots were generated to visualize the clustering of various cell types, including malignant cells, fibroblasts, T cells, and macrophages. The distribution of *ERP44* expression levels across these cell types was visualized using box plots and bar plots to determine the proportion of cells expressing *ERP44* in each identified cell type.

### Chemotherapy sensitivity analysis

The correlation between *ERP44* expression and chemotherapy sensitivity was analyzed using data from the GDSC (Genomics of Drug Sensitivity in Cancer) database.^4^ The sensitivity to chemotherapeutic agents, including Paclitaxel, Vinblastine, Gemcitabine, Sorafenib, and Rapamycin, was evaluated by calculating the IC50 values and performing Spearman’s correlation analysis (|R| >0.10, FDR-adjusted p < 0.05) between *ERP44* expression and drug sensitivity. A p-value threshold of 0.05 was considered significant for the correlation analysis.

### Pan-cancer analysis of *ERP44* expression

A pan-cancer analysis of *ERP44* expression was performed using RNA-seq data from 33 cancer types in TCGA. Box plots were generated to compare *ERP44* expression (log2[TPM+1] values) between tumor and normal tissues, with statistical significance assessed using Wilcoxon signed-rank tests (FDR-adjusted p < 0.01). For survival analysis, patients were dichotomized into high/low ERP44 expression groups based on median expression in each cancer type. A forest plot was created to display univariate Cox regression results (HR with 95% confidence intervals) for overall survival across all 33 cancers, with statistical significance threshold set at p < 0.05 (two-sided).

### Validation of *ERP44* expression

The human oral squamous cell carcinoma line SCC15 and the normal human immortalized oral epithelial cell (HIOEC) line were obtained from Otwo Biotech (Shenzhen, China). Both cell lines were maintained in RPMI-1640 medium (Life Technologies, Shanghai) under standard culture conditions: 37°C, 5% CO_2_, and high humidity. Total RNA extraction was performed using Thermo Fisher’s RNA isolation reagent, followed by cDNA synthesis with the PrimeScript RT Kit (Thermo Fisher). Gene expression analysis was carried out via quantitative RT-PCR (qRT-PCR) on an Applied Biosystems ABI 7900HT platform, with relative expression levels calculated using the 2^−ΔΔCT^ method.

### Statistical analysis

All statistical analyses were conducted using R (version 4.1.0). P-values less than 0.05 were considered statistically significant. Data visualization was performed using the ggplot2, pheatmap, and forestplot packages.

## Results

### Identification of redox metabolism-related DEGs

**FIGURE 2:**
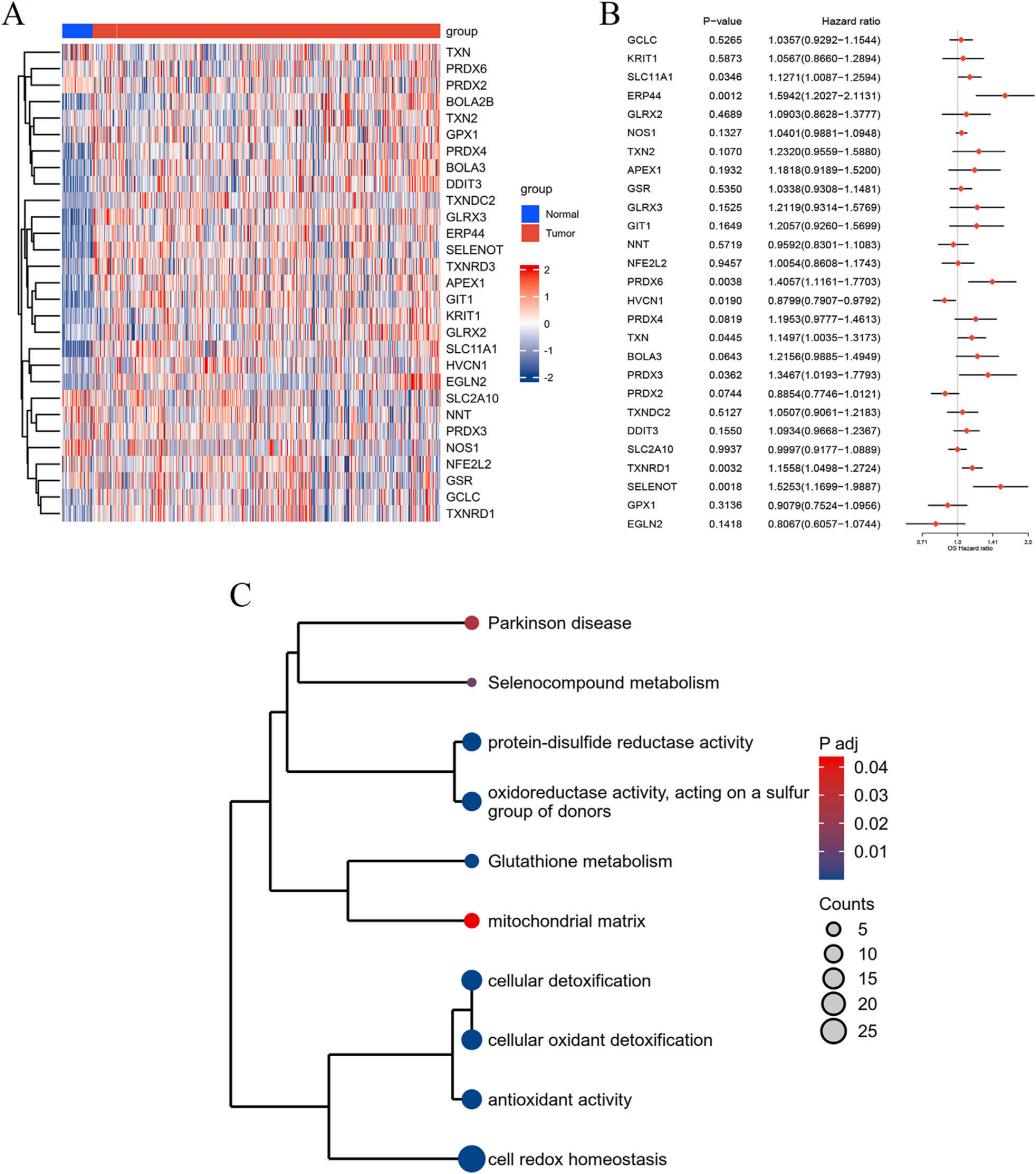
Differential expression and enrichment analysis of RMGs in HNSC. **(A)** Heatmap depicting the expression profiles of DEGs related to redox metabolism between normal (blue) and tumor (red) groups. Rows represent genes and columns represent individual samples. **(B)** Forest plot illustrating the results of the univariate Cox regression analysis of the identified DEGs. Hazard ratios (HRs) with their 95% confidence intervals (CIs) and p-values are shown for each gene. **(C)** Enrichment analysis of the redox metabolism-related DEGs, visualized as a clustered dendrogram. The nodes represent enriched terms, sized by gene count and colored by adjusted p-value, with significant associations to pathways and biological processes.

To unveil the differential expression of genes associated with redox metabolism between tumor and normal samples, we employed the limma package for comprehensive analysis. The resultant redox metabolism-related differentially expressed genes (DEGs) were visualized using a heatmap (Figure 2A), demonstrating distinctive expression profiles across the groups. The heatmap clearly indicates a set of genes exhibiting upregulated (in red) and downregulated (in blue) expression levels in the 548 tumor samples compared to the 44 adjacent normal samples. To assess the prognostic value of the identified DEGs, we performed univariate Cox regression analysis. The analysis results were illustrated in a forest plot, showcasing the hazard ratios (HRs) with their corresponding 95% confidence intervals (CIs) and p-values (Figure 2B). Several DEGs, such as *ERP44*, *PRDX6*, *TXNRD1*, and *SELENOT*, showed significant associations with patient survival (p < 0.05), warranting further investigation into their potential as prognostic biomarkers in HNSC. For further biological interpretation, enrichment analysis of the identified redox metabolism-related DEGs was conducted. The analysis revealed significant enrichment in pathways and processes crucial for cellular redox balance and oxidative stress management. Specifically, the DEGs were significantly associated with pathways including Parkinson’s disease, selenocompound metabolism, protein-disulfide reductase activity, oxidoreductase activity acting on a sulfur group of donors, glutathione metabolism, mitochondrial matrix, cellular detoxification, cellular oxidant detoxification, antioxidant activity, and cell redox homeostasis (Figure 2C). Overall, these analyses delineate a comprehensive profile of redox metabolism-related alterations in HNSC, underscoring their potential roles in disease progression and prognosis.

### Cox regression analysis of redox metabolism-related DEGs in HNSC patients

To determine the prognostic value of redox metabolism-related DEGs, we performed Cox regression analyses (Supplementary Figure S1). Univariate Cox regression analysis of the TCGA data indicated that ERP44 (p = 0.001), among other redox-related DEGs, exhibited the strongest association with poor prognosis in HNSC patients and was thus prioritized for further investigation. Additionally, age (p = 0.019), gender (p = 0.048), and metastasis status (M stage) (p = 0.002) were also found to be significant predictors of patient outcomes. Subsequent multivariate Cox regression analysis confirmed the independent prognostic value of *ERP44* (p = 0.003). Notably, ERP44 retained significance after adjusting for clinical covariates, underscoring its robustness as a candidate biomarker. Age (p = 0.028) and M stage (p = 0.010) remained significant in the multivariate model, while other clinical factors such as grade, stage, T stage, and N stage did not show significant prognostic value in the multivariate analysis.

### Prognostic and expression analysis of *ERP44*

**FIGURE 3:**
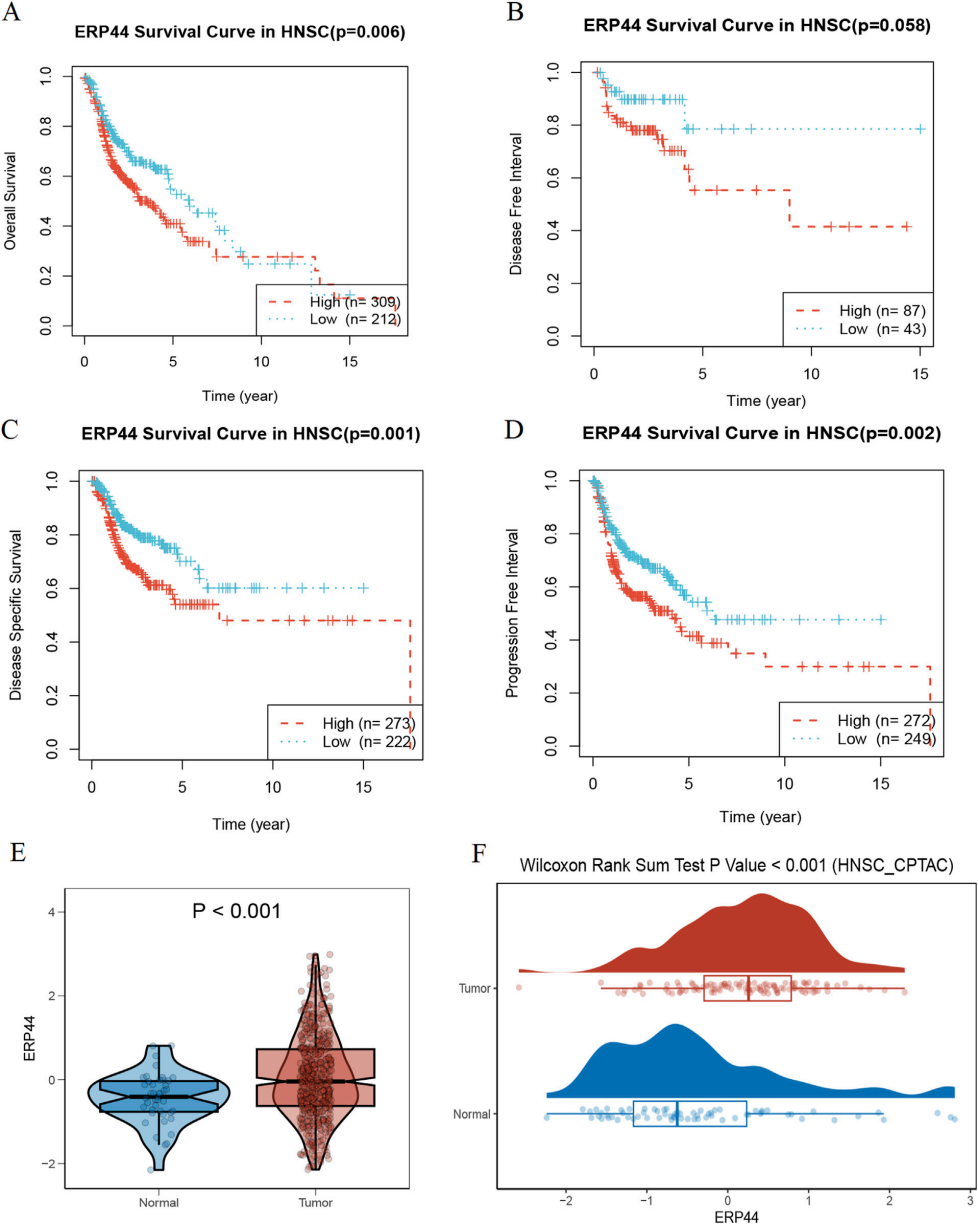
Prognostic and expression analysis of *ERP44* in HNSC. Kaplan-Meier survival curves analyzing the prognostic value of *ERP44* expression in the TCGA-HNSC dataset: **(A)** Overall Survival (OS), **(B)** Disease-Free Interval (DFI), **(C)** Disease-Specific Survival (DSS), and **(D)** Progression-Free Interval (PFI). **(E)** Violin plot displaying significantly higher *ERP44* gene expression in tumor tissues compared to normal tissues in the TCGA-HNSC dataset. **(F)** Box plot combined with a density plot showing significantly elevated *ERP44* protein expression in tumor tissues compared to normal tissues in the HNSC-CPTAC dataset (Wilcoxon Rank Sum Test p < 0.001).

**FIGURE 4:**
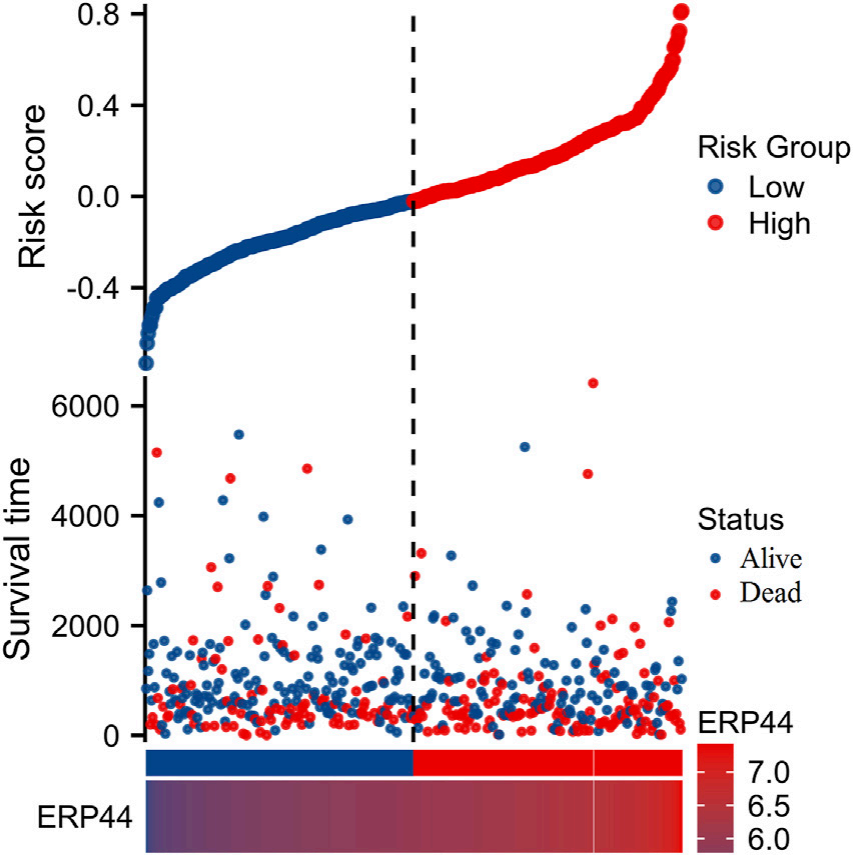
Risk factor analysis based on *ERP44* expression in TCGA-HNSC dataset. The upper plot shows the risk scores of patients, categorized into low-risk (blue) and high-risk (red) groups with the vertical dashed line as the demarcation. Survival time is plotted on the y-axis, indicating longer survival in the low-risk group. Patient status (alive or deceased) is marked with blue and red dots, respectively. The lower heatmap displays *ERP44* expression levels, with high expression depicted in lighter colors and low expression in darker colors.

Figure 3 illustrates the prognostic significance and expression analysis of *ERP44* in HNSC. Kaplan-Meier survival analyses based on TCGA-HNSC data revealed that high *ERP44* expression is significantly associated with reduced overall survival (OS) (Figure 3A, p = 0.006), disease-specific survival (DSS) (Figure 3C, p = 0.001), and progression-free interval (PFI) (Figure 3D, p = 0.002). Although a similar trend was observed for disease-free interval (DFI), the association was not statistically significant (Figure 3B, p = 0.058). Gene expression analysis demonstrated that *ERP44* expression levels were significantly higher in tumor tissues compared to normal tissues within the TCGA-HNSC dataset (Figure 3E, p < 0.001). Protein expression analysis using the HNSC-CPTAC dataset further corroborated these findings, showing significantly elevated protein levels of *ERP44* in tumor tissues compared to normal tissues, as determined by a Wilcoxon rank sum test (Figure 3F, p < 0.001). In addition, our cell experiments further confirmed that the *ERP44* gene was significantly upregulated in SCC15 cells (Supplementary Figure S2). As shown in Figure 4, our data reveal that patients classified in the low-risk group generally exhibit lower risk scores and a higher survival probability compared to those in the high-risk group. The survival status is indicated with blue dots for alive patients and red dots for deceased patients. It is apparent that a higher number of deceased patients (red dots) are found in the high-risk group, suggesting a negative correlation between higher risk scores and patient survival. Additionally, the heatmap below the plot illustrates *ERP44* expression levels, with color gradients from blue (lower expression) to red (higher expression). Patients in the high-risk group predominantly show higher *ERP44* expression levels, whereas those in the low-risk group exhibit lower *ERP44* expression. This inverse relationship between *ERP44* expression and survival underscores the potential prognostic value of *ERP44* in HNSC. In addition, Supplementary Figure S3 presents the expression levels of *ERP44* across various clinical subgroups within the TCGA-HNSC dataset. These findings indicate that *ERP44* expression varies significantly with tumor grade and treatment outcome. These results collectively underscore the potential role of elevated *ERP44* expression as a biomarker for poor prognosis in HNSC patients.

### GSVA enrichment analysis of *ERP44*

**FIGURE 5:**
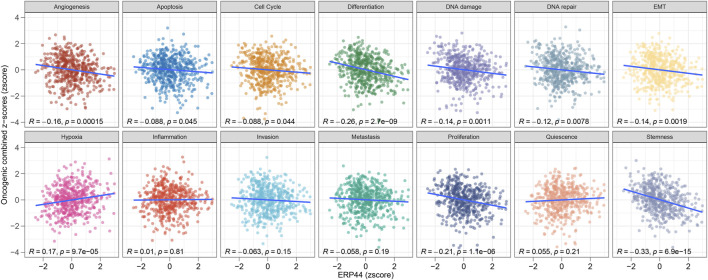
GSVA enrichment analysis of *ERP44* in the TCGA-HNSC dataset. Scatter plots illustrate the correlation between *ERP44* expression (z-score) and various oncogenic processes (combined z-scores).

Figure 5 illustrates the GSVA of *ERP44* in the TCGA-HNSC dataset, assessing the relationship between *ERP44* expression and various oncogenic pathways. Significant negative correlations were observed between *ERP44* expression and key oncogenic processes such as angiogenesis (R = −0.16, p = 0.00015), differentiation (R = −0.26, p = 2.7e-09), DNA damage (R = −0.14, p = 0.0011), DNA repair (R = −0.12, p = 0.0078), epithelial-mesenchymal transition (EMT) (R = −0.14, p = 0.0019), proliferation (R = −0.21, p = 1.1e-06), and stemness (R = −0.33, p = 6.9e-15). Conversely, a significant positive correlation was found between *ERP44* expression and hypoxia (i.e., elevated ERP44 levels corresponded to higher hypoxic conditions) (R = 0.17, p = 9.7e-05). These results suggest that high *ERP44* expression is associated with decreased activity in key oncogenic pathways such as angiogenesis, cell cycle, and differentiation, while showing a positive association with hypoxia in HNSC. This highlights the multifaceted role of *ERP44* in the modulation of tumor-related processes.

### Evaluation of immune cell infiltration and correlation with *ERP44* expression

**FIGURE 6:**
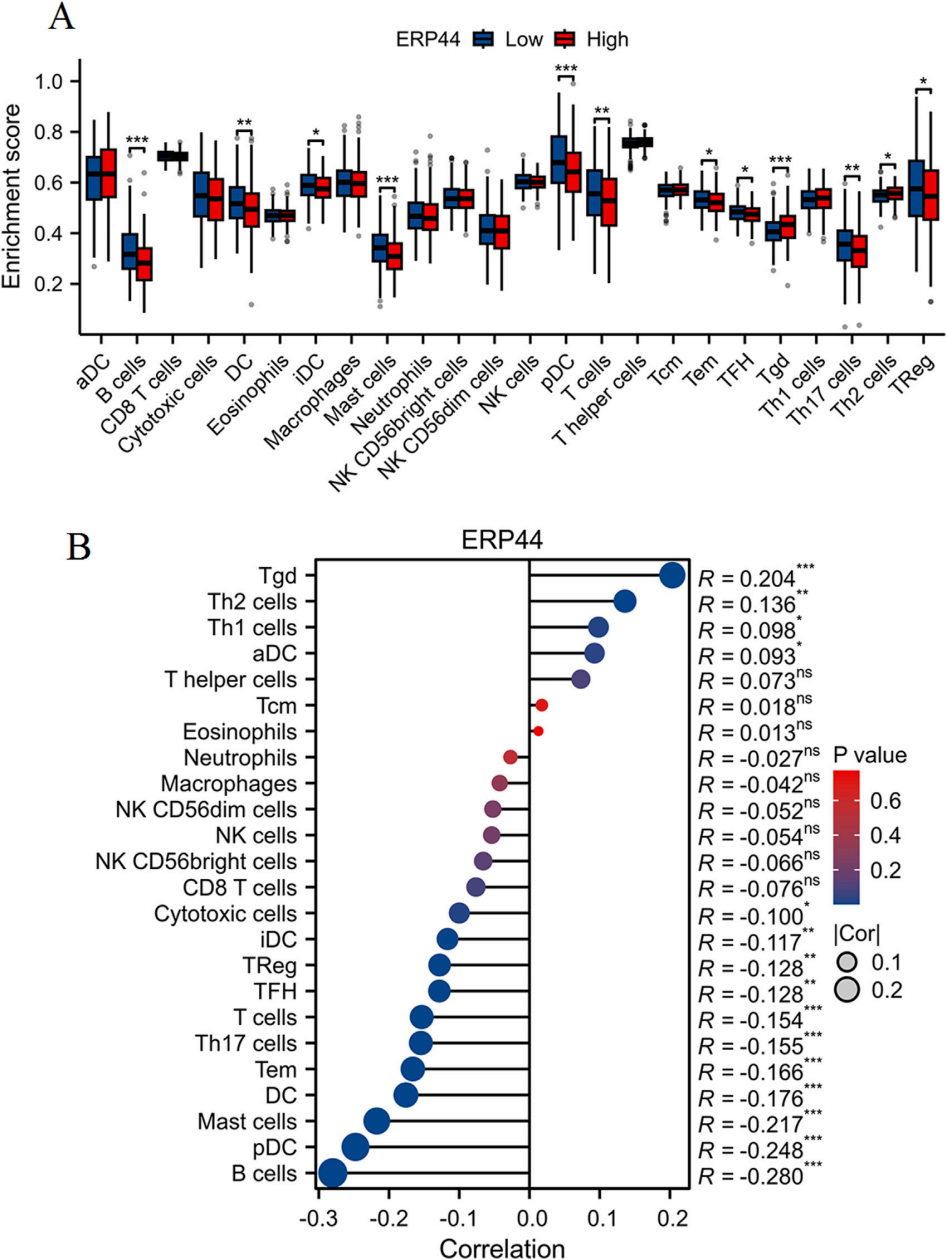
Evaluation of immune cell infiltration and correlation with *ERP44* expression. **(A)** Box plots showing the enrichment scores of various immune cell types between high (red) and low (blue) *ERP44* expression groups. **(B)** Correlation analysis between *ERP44* expression and immune cell infiltration levels. *p < 0.05, **p < 0.01, ***p < 0.001.

As shown in Figure 6A, the box plots illustrate significant differences in immune cell infiltration levels between the high and low *ERP44* expression groups. Notably, higher *ERP44* expression is associated with increased infiltration of several immune cell types, including Tgd and Th2 cells (p < 0.05). Conversely, lower *ERP44* expression shows a significant association with increased infiltration of B cells, DC, mast cells, pDC, T cells, Th17 cells, and regulatory T cells (TReg) (p < 0.05). As shown in Figure 6B, the correlation analysis reveals that *ERP44* expression is positively correlated with Th2 cells (R = 0.204, p < 0.001) and Th1 cells (R = 0.136, p < 0.01). Negative correlations were observed between *ERP44* expression and B cells (R = −0.280, p < 0.001), pDC (R = −0.248, p < 0.001), mast cells (R = −0.217, p < 0.001), and Th17 cells (R = −0.176, p < 0.001). These results suggest that *ERP44* expression in HNSC is associated with distinct patterns of immune cell infiltration, implicating its potential role in the tumor immune microenvironment.

### Single-cell analysis of *ERP44* gene expression

**FIGURE 7:**
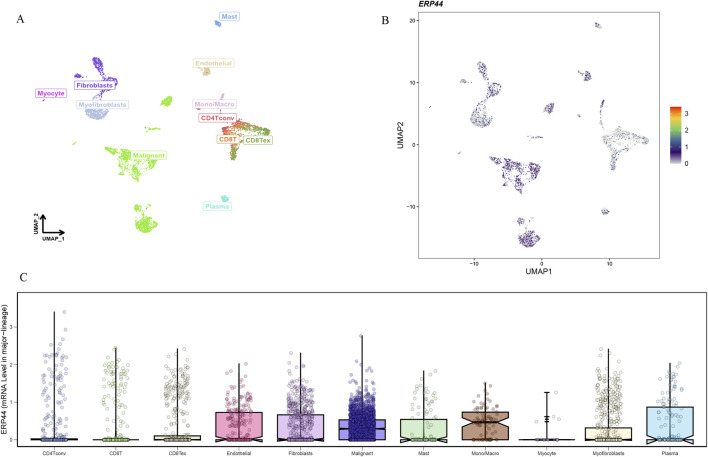
Single-cell analysis of *ERP44* gene expression in the GSE103322 dataset. **(A)** UMAP plot displaying the clustering of various cell types. **(B)** UMAP plot showing *ERP44* gene expression across different cell clusters, with the color gradient indicating the expression level. **(C)** Box plot illustrating the distribution of *ERP44* mRNA expression levels within each cell type.

**FIGURE 8:**
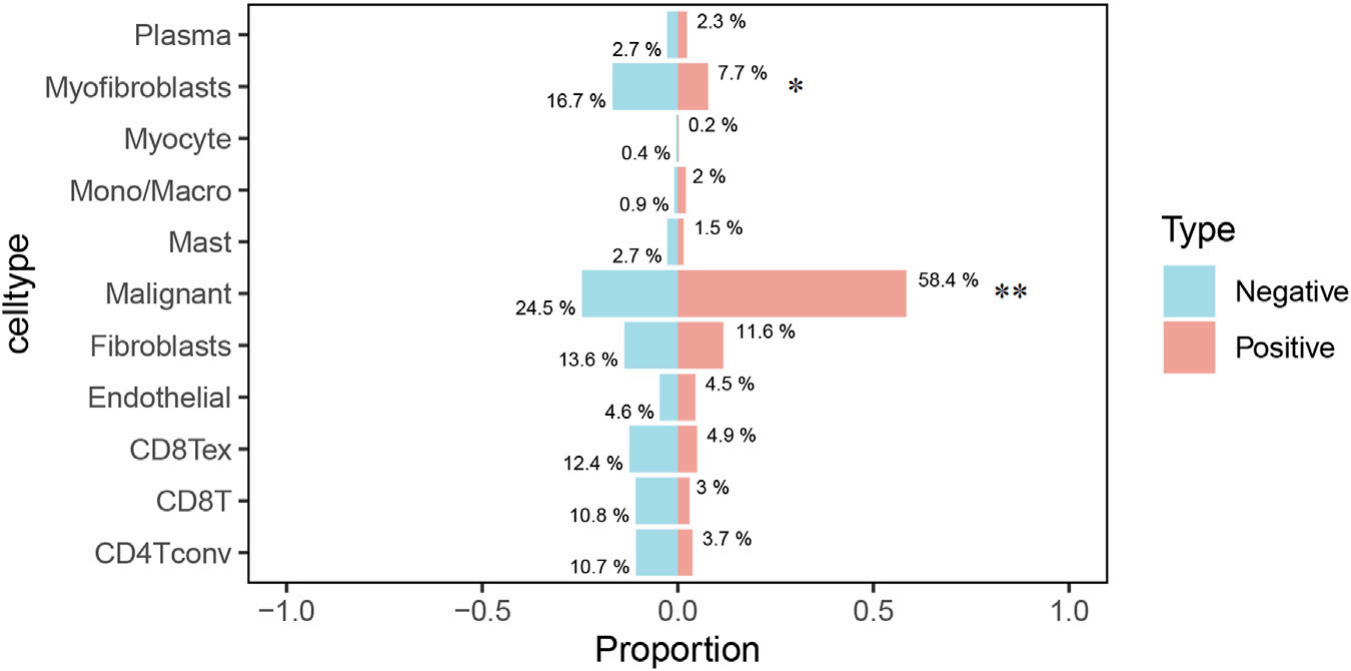
The bar plot illustrates the proportion of cells expressing ERP44 (positive, red) versus not expressing ERP44 (negative, blue) within each identified cell type. *p < 0.05, **p < 0.01.

The Uniform Manifold Approximation and Projection (UMAP) plot shows the clustering of different cell types, including CD4 T cells (CD4Tconv), CD8 T cells (CD8T and CD8Tex), endothelial cells, fibroblasts, malignant cells, mast cells, monocytes/macrophages (Mono/Macro), myocytes, myofibroblasts, and plasma cells (Figure 7A). The UMAP plot for *ERP44* expression indicates variation in *ERP44* expression levels across the identified cell clusters, with a color gradient representing the expression level (Figure 7B). As shown in Figure 7C, a box plot further details the distribution of *ERP44* mRNA expression levels within each cell type. The analysis reveals that *ERP44* is differentially expressed among the various cell types, with notably higher expression in malignant cells and fibroblasts compared to immune cells such as CD4 T cells, CD8 T cells, and plasma cells. In addition, the bar plot shows the proportion of cells expressing *ERP44* (positive, red) versus not expressing *ERP44* (negative, blue) within each identified cell type. Notably, malignant cells exhibited the highest proportion of *ERP44* expression, with 58.4% of malignant cells being ERP44-positive compared to 24.5% negative (p < 0.01). Myfibroblasts also showed a significant expression of *ERP44*, with 7.7% positive compared to 16.7% negative (p < 0.05) (Figure 8). These findings indicate that *ERP44* is differentially expressed across various cell types in HNSC, with particularly elevated expression in malignant cells and fibroblasts, suggesting its potential role in tumorigenesis and the tumor microenvironment.

### Chemotherapeutic drug sensitivity

**FIGURE 9:**
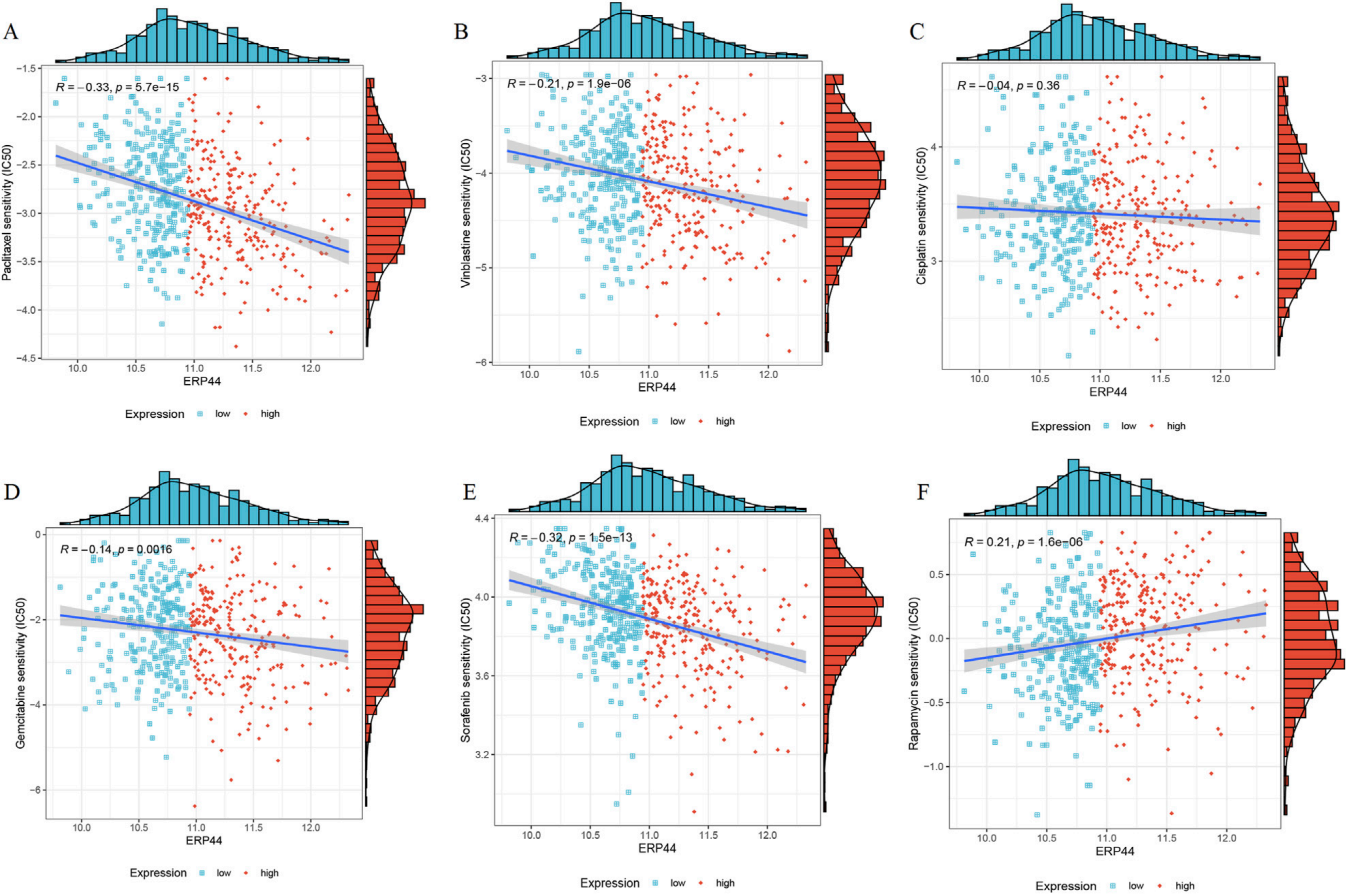
Chemotherapeutic drug sensitivity in relation to *ERP44* expression in the TCGA-HNSC dataset. Scatter plots and histograms illustrate the correlation between *ERP44* expression and the IC50 values of various chemotherapeutic drugs, including Paclitaxel **(A)**, Vinblastine **(B)**, Cisplatin **(C)**, Gemcitabine **(D)**, Sorafenib **(E)**, Rapamycin **(F)**. Blue points represent low *ERP44* expression, and red points represent high *ERP44* expression.

A significant negative correlation was observed between *ERP44* expression and Paclitaxel sensitivity (R = −0.33, p = 5.7e-15), suggesting that higher *ERP44* expression is associated with increased sensitivity to Paclitaxel (Figure 9A). Vinblastine sensitivity also showed a significant negative correlation with *ERP44* expression (R = −0.21, p = 1.9e-06), indicating greater sensitivity to Vinblastine in the high *ERP44* expression group (Figure 9B). No significant correlation was found between *ERP44* expression and Cisplatin sensitivity (R = −0.04, p = 0.38) (Figure 9C). A slight negative correlation was noted between *ERP44* expression and Gemcitabine sensitivity (R = −0.14, p = 0.016), implying that higher *ERP44* expression may lead to increased sensitivity to Gemcitabine (Figure 9D). Sorafenib sensitivity showed a strong negative correlation with *ERP44* expression (R = −0.32, p = 1.5e-13), indicating higher sensitivity to Sorafenib in tumors expressing higher levels of *ERP44* (Figure 9E). Contrarily, Rapamycin sensitivity exhibited a significant positive correlation with *ERP44* expression (R = 0.21, p = 1.6e-06), suggesting that higher *ERP44* expression correlates with decreased sensitivity to Rapamycin (Figure 9F). These results suggest that *ERP44* expression levels are significantly associated with the sensitivities to several chemotherapeutic drugs, including Paclitaxel, Vinblastine, Gemcitabine, Sorafenib, and Rapamycin, highlighting the potential of *ERP44* as a predictive biomarker for chemotherapy response in HNSC.

### Pan-cancer analysis of *ERP44*

**FIGURE 10:**
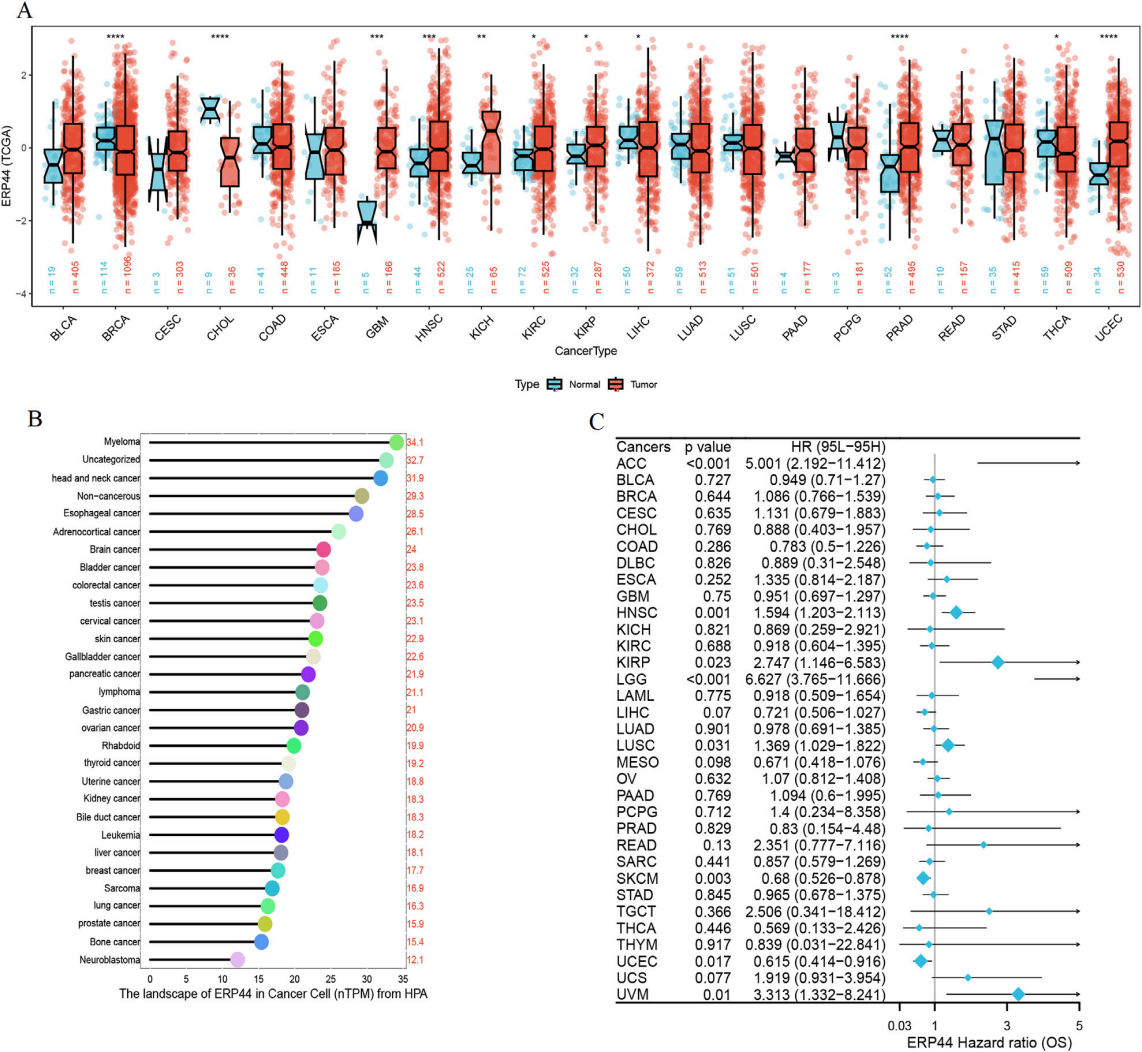
Pan-cancer analysis of *ERP44* expression. **(A)** Box plots illustrating *ERP44* expression in tumor tissues (red) versus normal tissues (blue) across various cancer types in the TCGA dataset. **(B)** Bar plot from the Human Protein Atlas (HPA) showing the landscape of *ERP44* expression (nTPM) in various cancer cell types. **(C)** Forest plot depicting the hazard ratios (HR) for overall survival (OS) associated with *ERP44* expression in different cancers.

**FIGURE 11:**
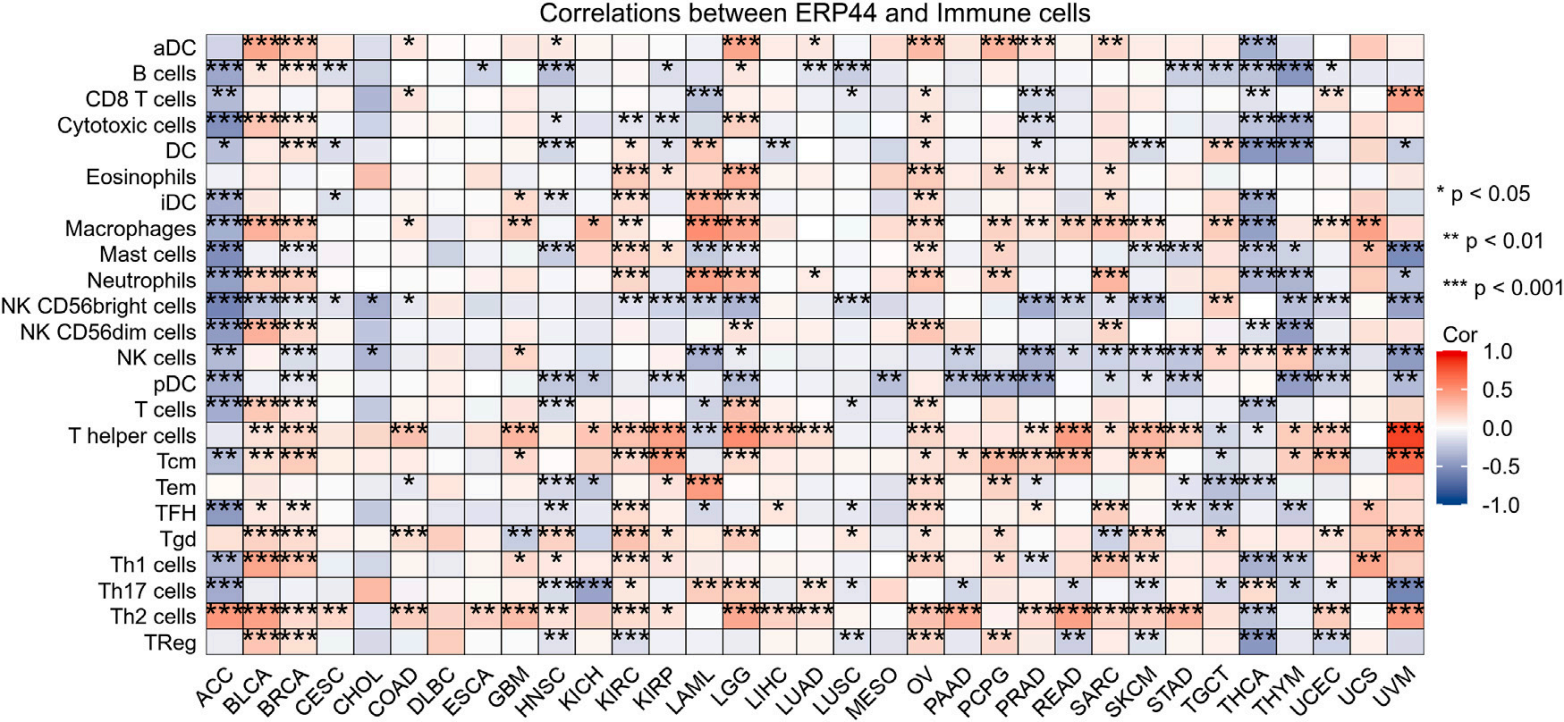
The heatmap displays the correlation between *ERP44* expression and the infiltration levels of various immune cell types across multiple cancer types.

Box plots illustrate the differential expression of *ERP44* between tumor tissues and normal tissues in multiple cancer types from the TCGA dataset. *ERP44* expression is significantly higher in tumor tissues compared to normal tissues in several cancers, including GBM, HNSC, KICH, KIRP, PRAD, and UCEC (p < 0.05) (Figure 10A). A bar plot from the Human Protein Atlas (HPA) highlights the landscape of *ERP44* expression in various cancer cell types, showing the highest expression in myeloma, followed by head and neck cancer, esophageal cancer, and others (Figure 10B). A forest plot displays the hazard ratios (HR) for overall survival (OS) associated with *ERP44* expression in different cancers. Significant associations were found in ACC (p < 0.001), HNSC (p = 0.001), KIRP (p = 0.023), LGG (p < 0.001), SKCM (p = 0.003), UCEC (p = 0.017), and UVM (p = 0.01) (Figure 10C). Collectively, these results underscore ERP44’s pan-cancer oncogenic role, where its overexpression correlates with poor survival across diverse malignancies. This establishes *ERP44* as a compelling biomarker for prognosis and a target for further mechanistic investigation in tumorigenesis. To delineate ERP44’s impact on tumor immunity, Figure 11 reveals its differential correlations with immune cell populations in a cancer-type-specific manner. For instance, significant positive correlations are noted between *ERP44* and activated dendritic cells (aDC) in several cancer types such as BLCA, BRCA, HNSC, and LGG, indicating a potential role in promoting immune response. Conversely, negative correlations are observed between *ERP44* expression and T regulatory cells (TReg) in cancers like HNSC, KIRC, and THCA, suggesting a possible inhibitory influence on immune suppression mechanisms. Similar nuanced relationships are observed with macrophages, B cells, neutrophils, and T cells. These findings illuminate ERP44’s multifaceted involvement in sculpting the tumor immune microenvironment. Its context-dependent interactions with immune infiltrates directly support its broader role in tumor progression and patient outcomes, offering mechanistic insights into how *ERP44* influences cancer biology and reinforcing its value as a therapeutic or prognostic biomarker.

## Discussion

Redox metabolism plays a pivotal role in the regulation of cellular functions, particularly in cancer progression. Cancer cells often exhibit altered redox homeostasis, which supports their survival, proliferation, and metastasis. A balanced redox environment is critical for maintaining normal cellular functions, while dysregulation can lead to oxidative stress, contributing to DNA damage, inflammation, and uncontrolled cell growth, all of which are hallmarks of cancer [14, 15]. Recent studies have emphasized that the perturbation of redox metabolism is not merely a consequence of cancer but an active driver of carcinogenesis [16, 17]. Moreover, the ability of tumor cells to adapt to oxidative stress via enhanced antioxidant defense mechanisms is essential for their survival in the hostile tumor microenvironment [18]. Thus, targeting redox metabolism-related genes offers a promising strategy for cancer therapy.

Our study identified a set of differentially expressed genes (DEGs) associated with redox metabolism in HNSC, with prominent candidates such as *ERP44*, showing significant prognostic potential. *ERP44*, an endoplasmic reticulum resident protein involved in disulfide bond formation and calcium homeostasis, has garnered increasing attention in cancer research [19]. Previous studies in HNSC have implicated *ERP44* in tumor progression; *ERp44*, secreted via exosomes from ER-stressed nasopharyngeal carcinoma cells, is a key contributor to chemoresistance in nasopharyngeal carcinoma [20]. The interaction between *ERp44* and ACLY facilitates NPC progression by regulating EMT [21]. Depletion of *ERp44* markedly impaired oral cancer cell proliferation and colony formation [22]. Beyond HNSC, *ERP44* dysregulation has been documented in diverse malignancies including gastric cancer, lung cancer, and gliomas, where it influences proliferation, apoptosis resistance, and endoplasmic reticulum stress [23–25]. The implications of these findings are twofold: first, they not only confirm the significance of *ERP44* overexpression in HNSC but also underscore the potential of these DEGs as biomarkers for disease progression and therapeutic response; second, they highlight the importance of redox-related metabolic pathways in the pathology of HNSC.

Previous studies have shown that dysregulation of redox metabolism is often associated with poor prognosis in various cancers. For example, the redox-associated genes *NCF2*, *VASN*, *FKBP1B*, and *TXNDC2* have been identified as central genes, potentially serving as a dependable prognostic indicator for the clinical management of glioma [13]. The genes associated with redox signatures may serve as indicators for forecasting the prognosis and evaluating the effectiveness of immunotherapy in individuals diagnosed with endometrial carcinoma [12]. Our findings reinforce these observations, particularly for *ERP44*, whose overexpression was associated with decreased OS, DSS, and PFI in HNSC patients. This alignment with prior research supports the notion that redox-related markers can serve as vital prognostic indicators, especially as tumors develop resistance to conventional treatments. The enrichment analysis of identified DEGs further elucidated key pathways related to redox metabolism, including processes vital for cellular detoxification, oxidative stress management, and metabolic reprogramming. The significant associations of the DEGs with pathways such as mitochondrial matrix regulation and glutathione metabolism provide insights into the adaptive changes that cancer cells undergo in response to oxidative stress [26, 27]. These findings support the premise that targeting redox homeostasis could represent a promising avenue for therapeutic intervention in HNSC.

An intriguing aspect of our comprehensive study is the meticulous evaluation of *ERP44* expression in relation to the infiltration of various immune cell types. Through our detailed analysis, we discovered a notable association indicating that high levels of *ERP44* expression correlated significantly with an increased infiltration of Tgd and Th2 cells, while conversely, lower expression levels were associated with an enhanced presence of TReg and other diverse immune cell types. This seemingly complex pattern, *ERP44* associating positively with Th2 cells (generally considered pro-tumorigenic and immunosuppressive) and negatively with TReg cells (also immunosuppressive), warrants deeper interpretation. Mechanistically, ERP44’s role in endoplasmic reticulum redox and calcium signaling could differentially influence immune cell recruitment or function. Its negative correlation with TRegs might reflect altered tumor cell signaling that limits TReg chemoattraction or survival. Conversely, its positive link with Th2 cells could be mediated through the promotion of a Th2-favoring cytokine milieu by ERP44-overexpressing tumor or stromal cells, potentially facilitating tumor immune evasion. This dualistic influence underscores ERP44’s context-dependent immunomodulatory potential within the TME. This observation is particularly compelling as it aligns with the emerging body of literature that suggests redox environments within tumors can profoundly modulate immune responses, thereby influencing the complex interactions between tumors and the immune system, which may ultimately affect outcomes in immunotherapy, as highlighted by previous studies [28–30]. Furthermore, the unique expression patterns of *ERP44* observed among different immune cell types could provide valuable insights into the intricate tumor immune microenvironment, thereby underscoring its potential as a promising therapeutic target in the ongoing quest for more effective cancer treatments. Our single-cell analysis revealed that *ERP44* is significantly expressed in malignant cells and fibroblasts, with minimal expression in immune cells. This fibroblast-specific expression pattern is significant, as cancer-associated fibroblasts (CAFs) are key architects of the TME and major contributors to immunosuppression and therapy resistance [31]. High *ERP44* in CAFs could influence extracellular matrix remodeling, cytokine secretion, or redox communication, thereby indirectly shaping immune cell infiltration and function. This finding suggests that *ERP44* may play a role not only in tumor cell biology but also in the tumor microenvironment, where interactions between malignant cells and surrounding stromal components can drive tumor progression and therapeutic resistance [32, 33]. These findings are consistent with research carried out in various other forms of cancer, underscoring the significant influence of stromal elements in altering tumor behavior and their responsiveness to treatment options [34].

The assessment of chemotherapeutic drug sensitivity in relation to *ERP44* expression has provided compelling evidence for its predictive value, shedding light on the intricate relationship between this protein and the efficacy of various cancer treatments. Our results indicated that higher levels of *ERP44* expression were significantly associated with increased sensitivity to well-known chemotherapeutic agents such as Paclitaxel and Vinblastine, both of which are commonly utilized in treatment regimens for HNSC. Conversely, we observed that elevated expression of *ERP44* correlated with a decreased sensitivity to Rapamycin, an mTOR inhibitor. This suggests that ERP44-overexpressing tumor cells might possess enhanced survival mechanisms that bypass mTOR dependence, potentially related to its roles in ER stress adaptation or calcium signaling. The increased sensitivity to microtubule-targeting agents (Paclitaxel, Vinblastine) could arise from ERP44-dependent alterations in cellular stress responses or protein folding pathways crucial for surviving mitotic disruption. Such contrasting relationships suggest that *ERP44* may hold considerable potential not only as a prognostic marker but also as a valuable predictor of chemotherapeutic response, offering insights that could enhance personalized treatment approaches. This notion aligns with previous reports that emphasize the urgent need for reliable biomarkers capable of guiding treatment strategies in an increasingly complex and multifaceted therapeutic landscape, where the selection of the most effective treatment can significantly impact patient outcomes [35–37].

While our findings offer significant insights into the complex landscape of HNSC, it is essential to acknowledge both the novelty and limitations inherent in this study. The integration of multi-dimensional data, encompassing everything from gene expression profiling to immune cell infiltration and drug sensitivity, provides a remarkably comprehensive view of *ERP44*’s multifaceted role in HNSC. However, it is crucial to note that further validation in larger, independent cohorts will be necessary to confirm the prognostic and predictive relevance of *ERP44* in clinical settings, ensuring that our findings can be reliably translated into therapeutic strategies. Additionally, while our study successfully identified several pathways that are enriched in redox metabolism-related DEGs, it is imperative that functional studies are conducted to elucidate the mechanistic underpinnings of these associations and their potential influence on tumor biology, thereby deepening our understanding of the intricate interactions at play within the tumor microenvironment.

## Conclusion

In conclusion, our findings together highlight the critical importance of redox metabolism in the progression and prognosis of HNSC. The identification of *ERP44* as a key player in this interplay underscores its potential utility as a biomarker and therapeutic target. By integrating redox metabolism pathways into the context of cancer biology, we move toward more personalized and effective treatment strategies, ultimately improving outcomes for HNSC patients.

## Data Availability

The original contributions presented in the study are included in the article/Supplementary Material, further inquiries can be directed to the corresponding author.

## Footnotes

1
https://portal.gdc.cancer.gov/

2
https://ualcan.path.uab.edu/analysis-prot.html

3
https://www.gsea-msigdb.org/gsea/msigdb

4
https://www.cancerrxgene.org/
