# Supplementary material for: Prognostic significance and therapeutic implications of redox metabolism-related genes in head and neck squamous cell carcinoma
Source: Exp Biol Med (Maywood). 2025 Sep 19;250:10623. doi: 10.3389/ebm.2025.10623 (PMC12492447; doi:10.3389/ebm.2025.10623)
Supplement: Supplementary file 1 [file Supplementaryfile1.docx]

Supplementary figures


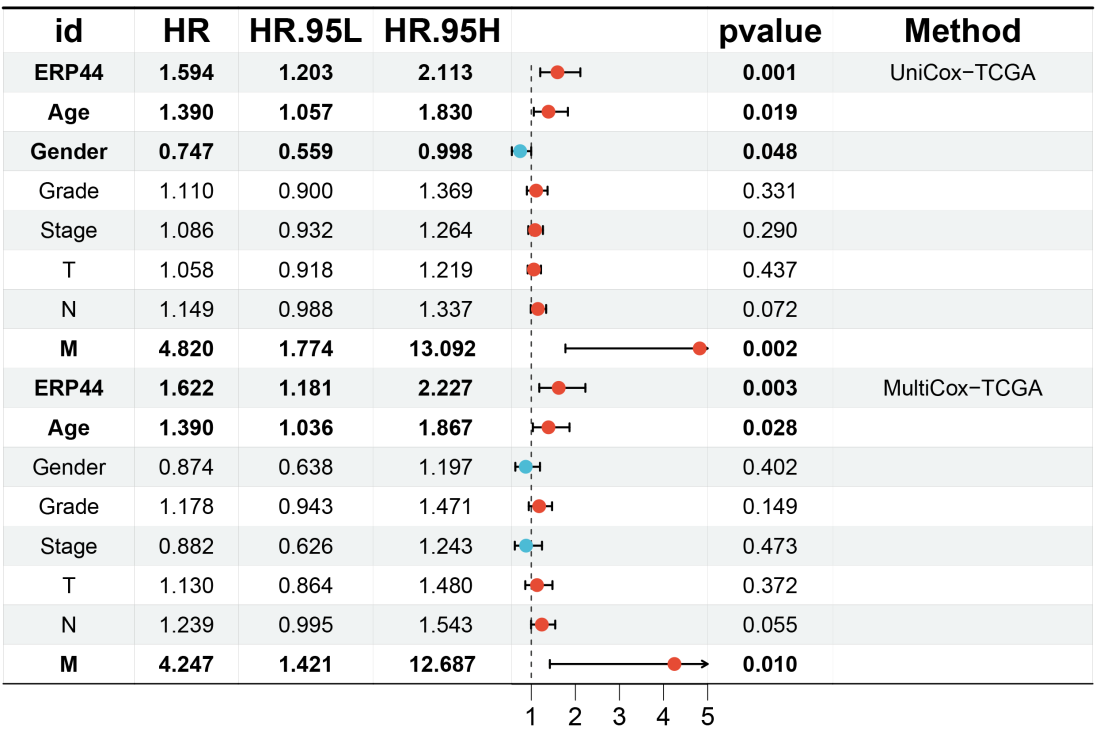


Figure S1 Cox regression analysis of redox metabolism-related DEGs.


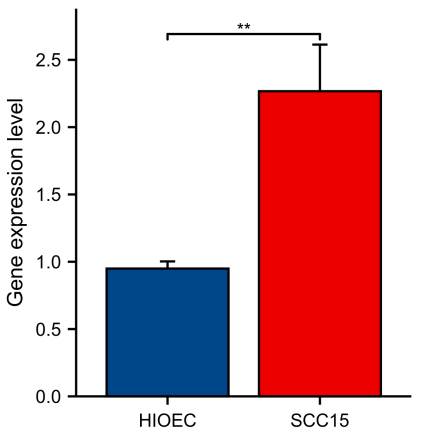


Figure S2 The ERP44 gene expression level was validated using cell experiments. **p < 0.01. T-test was applied to compare the differences between the two groups.


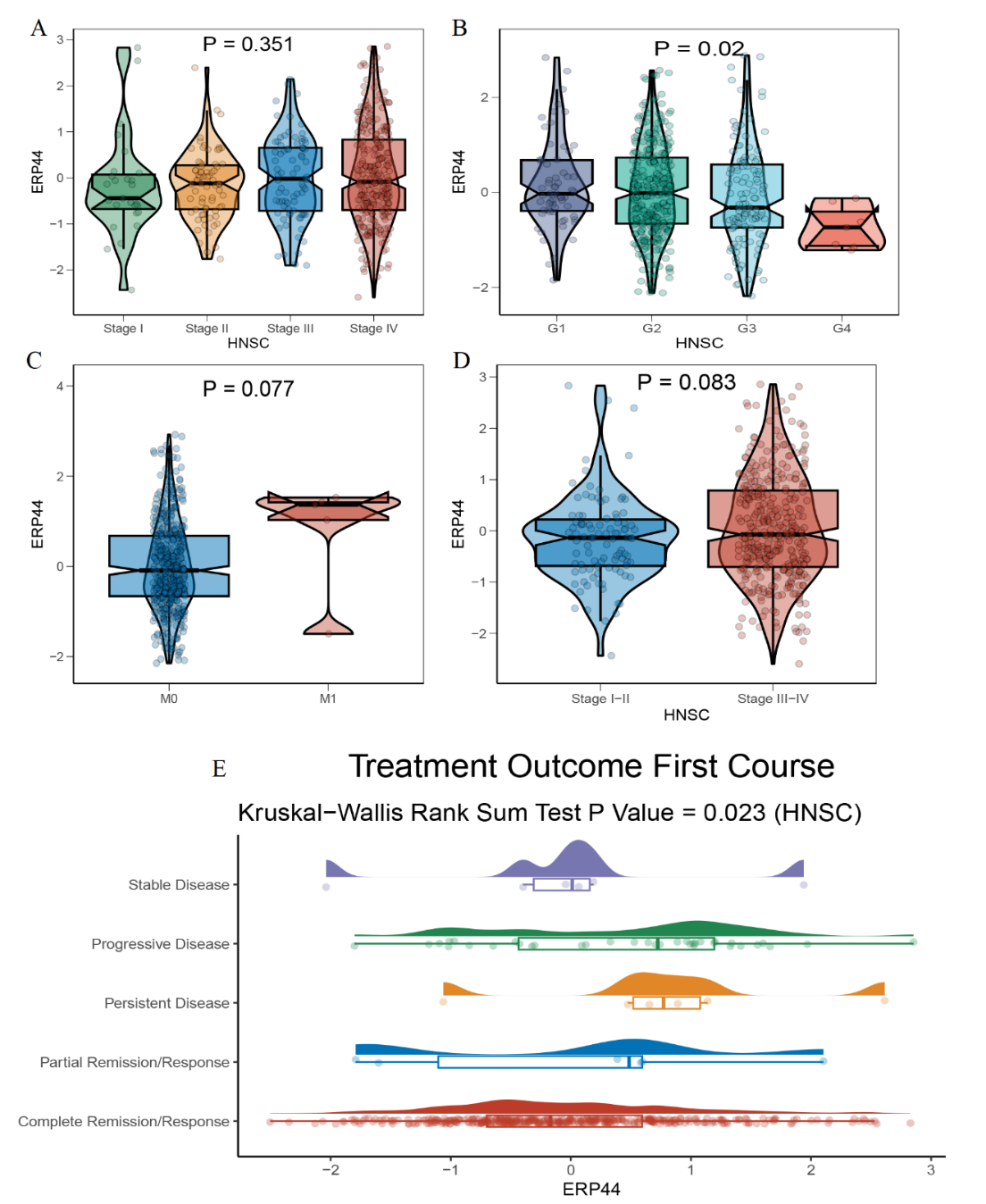


Figure S3 Analysis of ERP44 expression across clinical subgroups in the TCGA-HNSCC dataset. (A) Expression of ERP44 across different tumor stages (I-IV). (B) Significant differences in ERP44 expression were observed across tumor grades (G1-G4). (C) ERP44 expression was slightly higher in patients with metastasis (M1) compared to those without metastasis (M0), but the difference was not statistically significant (p = 0.077). (D) No significant differences in ERP44 expression were found between early (Stage I-II) and advanced stages (Stage III-IV) of HNSCC (p = 0.083). (E) Significant variation in ERP44 expression was observed across different treatment response groups (p = 0.023), with varying levels of expression in patients with stable disease, progressive disease, and different remission statuses.
