# Supplementary material for: Prognostic significance and therapeutic implications of redox metabolism-related genes in head and neck squamous cell carcinoma
Source: Exp Biol Med (Maywood). 2025 Sep 19;250:10623. doi: 10.3389/ebm.2025.10623 (PMC12492447; doi:10.3389/ebm.2025.10623)
Supplement: Supplementary file 2 [file Table1.docx]

Table S1 The clinical characteristics of patients with HNSCC in the TCGA-HNSCC dataset.

| Characteristics | n = 548 | Percentage |
| --- | --- | --- |
| Pathologic T stage |  |  |
| T1 | 49 | 8.94% |
| T2 | 140 | 25.55% |
| T3 | 101 | 18.43% |
| T4 | 176 | 32.12% |
| Pathologic N stage |  |  |
| N0 | 179 | 32.66% |
| N1 | 68 | 12.41% |
| N2&N3 | 182 | 33.21% |
| Pathologic M stage |  |  |
| M0&M1 | 193 | 35.22% |
| Pathologic stage |  |  |
| Stage I | 27 | 4.93% |
| Stage II | 73 | 13.32% |
| Stage III | 82 | 14.96% |
| Stage IV | 272 | 49.64% |
| Gender |  |  |
| Female | 143 | 26.09% |
| Male | 386 | 70.44% |
| Race |  |  |
| Asian | 11 | 2.01% |
| Black or African American | 48 | 8.76% |
| White | 453 | 82.66% |
| Age |  |  |
| <= 60 | 261 | 47.63% |
| > 60 | 267 | 48.72% |
| Histologic grade |  |  |
| G1 | 64 | 11.68% |
| G2 | 311 | 56.75% |
| G3&G4 | 132 | 24.09% |
| Alcohol history |  |  |
| No | 166 | 30.29% |
| Yes | 352 | 64.23% |
| Smoker |  |  |
| No | 122 | 22.26% |
| Yes | 394 | 71.90% |
